# Surgery

**Published:** 1896-06

**Authors:** C. A. Morton


### SURGERY.

We are all interested in **appendicitis,** whether we practise medicine or surgery. Every now and again we are called to the bedside of a patient suffering from this disease, and we have to take the grave responsibility of deciding whether operative interference is required. If we relieve symptoms by opium and congratulate ourselves on a fall in temperature, we may conclude that all is well, when unfortunately all is not well, and the precarious barrier thrown out by Nature around the sloughing appendix, may just have given way, and general infection of the peritoneum—which we feel almost powerless to deal with when once it has started—have commenced. Or, again, a case which we watch day by day as one of localised appendicitis may really have infected the general peritoneum from the first; and while we are anxiously waiting for improvement, more and more surely is the disease fixing its fatal hold on the patient.

Since the discussion on appendicitis at the meeting of the Bath and Bristol Branch of the British Medical Association,[3] in January, 1894, many very valuable contributions to the literature of the subject have appeared. I intend only to refer to them in connection with the all-important question, **When should we operate?** And, first I must say that it seems to me that if we base our opinion on any classification of the forms of the disease, we shall not find that opinion of much value as we stand by the bedside of the patient; for we cannot in many—perhaps in most—cases tell from the symptoms the actual condition of the appendix, or what course the disease will run. Morris of New

[3] *Bristol M.-Chir. J.*, 1894, xii. 9, 68.

York puts this forcibly.[1] He says: "We may speak of the acute or chronic form, the form of endo-appendicitis, or of perforation . . . and so on indefinitely; but as endo-appendicitis may be present on Monday and perforating appendicitis may appear on Wednesday in the same case without our being able to state what Friday appendicitis may be like, we might classify these cases as 'Monday,' 'Wednesday,' and 'Friday' appendicitis. . . . There are no groups of symptoms which will allow us to make a rational prognosis as to the eventual outcome, or the prospective complications in any progressing case of appendicitis, and we must abandon the hope of having any such classification of symptoms for a guide in the future. . . . I speak, then, unequivocally, knowing that some patients are to die and others are to suffer unnecessarily because their advisers will believe themselves to be upon a prognostic track. There is but one rule to be followed, and that is to isolate an infected appendix as promptly as we would isolate a case of diphtheria and for practically the same reasons, viz.: the infected appendix will probably infect other structures, and the infected throat is likely to infect other throats." Thus strongly writes Morris, a surgeon of large experience in appendicitis. On what facts does he rest so decided an opinion? He says: "Statistics from a large number of observers give an average death-rate in the principal attack of appendicitis of about 15 per cent. under medical treatment,"[2]; whereas he believes a death-rate of 2 per cent. is too high in cases operated on in the first attack, before infection has extended beyond the confines of the appendix. He has himself operated on 100 cases of appendicitis. In 59 cases where the infection was limited to the immediate vicinity of the appendix, and involving in many cases extensive operative work on account of adhesions, there were no deaths. In 23 cases where collections of pus were walled off from the general peritoneal cavity, 5 died: 2 of septicæmia, 1 of acute suppurative nephritis, 1 of intestinal obstruction from adhesions which could not be separated at the time of the operation on account of the patient's condition, and only one, "weak from several months of septicæmia," died of shock. These statistics, compared with those of cases not operated on, show that in such hands as Morris's the operation is practically free from risk, whereas the risk of waiting is very considerable. No doubt some of these cases would have recovered without operation: but I think the more we see of cases of appendicitis, the more we feel that we cannot separate the cases which will terminate favourably from those which will end fatally; and that if we attempt to, we may see several patients recover—perhaps in succession,—and then we shall lose a case which seems going on as favourably as any of the others. Surely, then, Morris is right when he urges removal in

[1] *Lectures on Appendicitis.* 1895, p. 35. [2] *Op. cit.*, p. 36.

all cases where the symptoms are marked and do not at once subside, seeing that there is so little risk in the operation if skilfully performed. Such teaching will seem strange to those who are in the habit of waiting for evidence of a collection of pus as an indication for operation. Unfortunately, while they are waiting for such evidence the general cavity of the peritoneum may become fatally infected. We must early in the disease open the peritoneum, isolate the infected area with sponges or iodoform gauze, then break into and drain any purulent collection, and remove the diseased appendix if possible. McBurney, as long ago as 1892,[1] criticised Treves's view as to the desirability of waiting for pus to come towards the surface. He writes: "The safe way to open these deep-seated abscesses is to first deliberately open the peritoneal cavity and carefully note the relations of the parts. It is then a comparatively simple matter to cautiously unravel the tangled mass of organs—that is, it is much simpler and safer than to cut steadily onward from the blind side, and before one knows what the wall of the abscess is made up of. Then, before such a deep-seated abscess is actually opened, if the road that has been cleared to it is lined with carefully held sponges, a small opening can be made into the abscess and the pus caught on a sponge almost guttatim and harmlessly removed. This practice has been a common and successful one in New York for a considerable time. Cases of deep-seated abscess opened at an early period in this manner will very rarely, indeed, end in any other way than in rapid recovery." I have successfully operated on two cases in this way. I opened the abdomen, and found in each case a swelling formed of adherent coils around a collection of pus in the region of the appendix. No adhesion had taken place to the abdominal wall. I very cautiously broke into the abscess cavity by gently separating the coils after packing around with iodoform gauze and sponges, and, as McBurney describes, sponged out the pus "almost guttatim," draining by means of iodoform gauze from the bottom of the abscess cavity out of the abdominal incision. In one case the sloughed appendix was discharged a few days after operation; in the other, I neither saw nor knew that I felt the appendix at the time of operation, nor was it discharged as a slough. I cannot agree with McBurney that it is a simple matter to unravel "the tangled mass of organs." It seems to me that in some cases it may be very difficult to break into the mass of adherent coils and find the pus without lacerating the inflamed intestine. I felt sure there was pus in these two cases from the definite nature and persistence of the localised swelling, together with marked symptoms, though no fluctuation was present. In fact, it seems to me distinctly dangerous to press very firmly in any attempt to get fluctuation, for fear of rupturing a localised collection into the

[1] *Med. Rec.*, 1892, xli. 423.

general peritoneal cavity. With regard to the value of rectal examination in appendicitis, McBurney writes:[1] "In the early stages of appendicitis this method has no value whatever. Of course, those who are accustomed to allow abscesses to reach a large size, will in time find a bulging tumor low down in the pelvis. We had better devote our attention to learning the signs of suppurative appendicitis before the abscess has threatened the rectum."

Morris's success in cases where **general peritonitis had already set in** is truly marvellous. Out of 6 cases of intense general septic peritonitis, with the whole abdominal cavity bathed in pus, 5 recovered. Such success is unheard of in this country. I have by free irrigation and drainage recently saved a patient into whose general peritoneal cavity a large quantity of stinking pus had escaped from rupture of a pelvic abscess: but although I have operated in several cases of septic peritonitis, I have never saved one yet; and we operate with little expectation of saving them, though we feel we ought to give them the very slight chance which operation affords. Lockwood and a few other surgeons in this country have, however, been now and then successful. But American surgeons—Morris, Murphy, McBurney, and others—have apparently saved many of these cases. Looking at the fact that when once general septic peritonitis has set in our resources are of so little value, how important becomes the question of early operation!

**Can we tell at the outset whether we are dealing with a case of localised appendicitis or general peritonitis?** Murphy[2] considers that we cannot, and he records a case in point, where only the symptoms of appendicitis were present and the young man was walking about, but pus was found free in the peritoneal cavity. By early operation in this case life was saved. A careful study of Murphy's 141 cases[3] will show that whereas general septic peritonitis was found at the operation, the symptoms did not point to a condition of such gravity in several of the cases. In case 103, a week after the sudden onset of the attack, the temperature was 99.5°, pulse 90, and there was no collapse, but general septic peritonitis was present. In case 108, 31 hours after the onset of the symptoms, there was no collapse, the temperature was 99.7°, and pulse 80, but the peritoneal cavity was full of thin purulent fluid. Contrast with these case 83 in which no peritonitis existed, but the appendix contained pus. The patient was 13 years of age. Murphy thus describes the symptoms: "This is a particularly interesting case, the temperature keeping above 105° and the pulse 134 from the beginning of the attack up to the time of operation. Abdomen enormously tympanitic and tender. He presented all the so-called classical symptoms of general septic peritonitis." McBurney's

[1] *Loc. cit.*, p. 425. [2] *Med. News*, 1895, lxvi. 1.
[3] *J. Am. M. Ass.*, 1894, xxii. 302, 347, 387, 423.

experience has been the same.[1] He writes, with reference to a series of cases he operated on (to which reference will again be made) in which either pus was found free in the peritoneum or general peritonitis had already commenced: "The character of the case has usually, but not always, been appreciated by the wide-spread tenderness, high temperature, rapid pulse, increased vomiting, and positively ill look of the patient. In some of the cases, the signs have been only those of ordinary well-localised suppurative appendicitis." These cases of perforative appendicitis do not begin by collapse, as in the cases of perforating gastric ulcer. The symptoms at the onset may be the same in mischief within the appendix as in perforation with commencement of general peritonitis. The pain in perfectly localised appendicitis is often, at first, not in the right iliac fossa, but at the epigastrium; and the indication from general tympanitic distension and tenderness is not always a trustworthy one. I operated, a few weeks ago, on a case of appendicitis which had set up general suppurative peritonitis, in which the tympanitic distension was less than in many cases of localised mischief. The indication of general tenderness could not be relied on, as the boy had been placed fully under opium. The temperature is, of course, no guide, nor in the early stage the pulse either.

Murphy[2] is emphatic in his statement that all cases of appendicitis beginning with severe symptoms ought to be operated on. He says: "It is estimated on good authority that from 27 to 30 per cent. of all cases treated medicinally terminate fatally sooner or later, if not operated on." Out of his own 141 cases he lost 16: but in 12 of these septic peritonitis was present at the time of operation. He considers the dangers of the operation in competent hands to be *nil*.

Barling,[3] in "The Ingleby Lectures on Appendicitis," discusses the question of operation. He lays down rules for the differentiation of perforating appendicitis from other kinds, and only advises immediate operation when the former is present. But I think his indications will not stand the test of clinical work. He admits that in children we can hardly expect to differentiate perforation at all. McBurney[4] records several cases of **peritonitis from perforation of the appendix** saved by washing out with sterilised saline solution, and drainage by strands of gauze and a glass tube passed into the pelvis. He uses a sterilised saline solution, believing that this does not damage the endothelium of the peritoneum, as even mild antiseptic solutions do: for anything which damages the endothelium will interfere with absorption, a process of great importance if a patient suffering from septic peritonitis is to recover. I do not know that we can call all McBurney's cases general peritonitis, but they were all cases of general peritoneal

[1] *Med. Rec.*, 1895, xlvii. 385. [2] *J. Am. M. Ass.*, 1894, xxii. 302. [3] *Brit. M. J*, 1895, i. 1255. [4] *Loc. cit.*

infection. In some no evidence of the presence of peritonitis, on operation, is given, but in these purulent fluid had run down from the region of the appendix into the pelvis, and the onset of peritonitis was only a matter of time. His success in so many cases seems to me to lie not so much in the use of saline solutions and multiple gauze drains as in the fact that he operated within the first few days of the attack. I have lately operated in one case by McBurney's method. A boy was admitted into the Bristol Children's Hospital with symptoms of general peritonitis; within 36 hours of the onset (a few hours after his admission) I cut down on the appendix and found it sloughing and perforated, with a small collection of stinking pus around; and from the region of the pelvis (before I reached the pus around the appendix) purulent fluid escaped freely. After removing the appendix and swabbing out the localised collection of pus, I very thoroughly washed out the peritoneal cavity with saline solution (90 grs. to a quart), sterilised by boiling, and as there was remarkably little tympanitic distension, I was able to do this very thoroughly. I then drained from every direction with Morris's "wick" drains. For three days the boy's condition seemed encouraging; but although he never vomited again or seemed in pain, nor did tympanitic distension set in, yet after the first few days his strength gradually failed, and he died at the end of a week. At the necropsy pockets of pus were found here and there amongst the intestines, showing that the septic process had not been controlled.

The generally received view in this country is, that if operation for **relapsing appendicitis** is called for, it should be undertaken between the attacks, in the quiescent period. But if we run so considerable a risk as some American surgeons teach us we do in not operating on every severe case of appendicitis, what are the risks to the patient in not operating in cases in which the attack is a recurrent one? If we carefully examine Treves's record of 18 cases[1] of relapsing appendicitis on which he operated, we shall find that in several the appendix was not buried in adhesions, and might have caused fatal perforation at any time. In several it contained stinking pus, and one such, in which there had been eight attacks, had no adhesions at all. In several, collections of "custard-like material" were found amongst the adhesions, which could hardly have been other than drying-up pus. In an earlier record[2] of another series of these cases, he says that in the majority dealt with by operation pus was found. Hence it is evident that relapsing cases are not by any means free from the risk of setting up general peritonitis, and the advice usually given only to operate between the attacks cannot be safely followed. In Treves's first case of the first series, in the third attack "symptoms of general peritonitis ensued and the child's

[1] *Brit. M. J.*, 1895, i. 517. [2] *Ibid.*, 1893, i. 835.

life was despaired of." Three months later, when operation was undertaken (some tenderness persisting), he found a small encysted abscess and the appendix extensively ulcerated. This case shows how dangerous a relapsing case may be, and in connection with it I will refer to one under my own care. In the autumn I saw, in consultation, a gentleman with a swelling in the right iliac fossa. All the symptoms had subsided, but they had been such as to suggest appendicitis. I cut down on the swelling and found pus shut off from the general peritoneal cavity. As is the generally-accepted rule in such cases, and as I have found quite sufficient in other cases of the same nature I have operated on, I was content with the evacuation of the pus and left the appendix to take care of itself, as I could not feel it within the abscess cavity. In this case, for many weeks a sinus persisted, and several fæcal concretions, such as are found in the appendix, came away. At last it closed, but pus collected behind, and it discharged again. It finally closed about three months after operation, and the patient was able to get about and attend to his business. Then suddenly symptoms of general peritonitis set in, and although I operated within a few hours of my first seeing him and removed the perforated appendix, I did not succeed in saving his life. I know of one or two other cases where the mischief in the appendix has not come to an end after a localised collection of pus in connection with it has been drained and the sinus has closed. But this so rarely happens that probably we are not justified in laying open such localised collections of pus into the general peritoneal cavity by attempting to isolate and remove the infected appendix.

Mr. Treves[1] has just given us his present opinion as to the need of operation in appendicitis. He gives the mortality at 11 per cent. in non-suppurative cases, but 30 to 40 per cent. if abscess is present. But who can say in any given case that no abscess is present? As McBurney and others have shown, a collection of pus is frequently found long before fluctuation could be detected. Treves says: "An incision in perityphlitis is very seldom called for before the fifth day," and he strongly condemns what he calls "reckless and unnecessary incisions made without due cause during the first two or three days of the attack." We can only presume that many of the operations of McBurney, Murphy, and Morris would be thus described. Several years ago Treves wrote that he considered the indication for operation was distinct evidence of abscess, and to this he seems still to adhere. He says, however, that "in the ultra-acute cases it is well that the abdomen be opened at once in the cæcal region, and the case considered and dealt with as one of perforative peritonitis." But how impossible it is to be sure of the absence of perforative peritonitis in all severe cases I have already quoted instances to prove.

C. A. MORTON.

[1] *A System of Surgery.* 1896, II. 627.

